# Video Capsule Endoscopy Optimal Timing in Overt Obscure Gastrointestinal Bleeding

**DOI:** 10.3390/diagnostics12010154

**Published:** 2022-01-09

**Authors:** Joo Hye Song, Ji Eun Kim, Hwe Hoon Chung, Sung Noh Hong, Heejung Kim, Eun Ran Kim, Dong Kyung Chang, Young-Ho Kim

**Affiliations:** Department of Medicine, Samsung Medical Center, Sungkyunkwan University School of Medicine, Seoul 06351, Korea; joohye.song@samsung.com (J.H.S.); jieun05.kim@samsung.com (J.E.K.); hwehoon.chung@samsung.com (H.H.C.); erkim@samsung.com (E.R.K.); dkchang@skku.edu (D.K.C.); bowelkim@gmail.com (Y.-H.K.)

**Keywords:** overt obscure gastrointestinal bleeding, video capsule endoscopy, timing of procedure, detection

## Abstract

Video capsule endoscopy (VCE) has become the noninvasive diagnostic standard in the investigation of overt obscure gastrointestinal bleeding (OGIB), with a high positive and negative predictive value. However, the diagnostic yield of the VCE is thought to depend on when it was performed. We evaluate the optimal timing performing VCE relative to overt OGIB to improve the diagnostic yield. A total 271 patients had admitted and underwent VCE for overt OGIB between 2007 and 2016 in Samsung Medical Center, Seoul, Korea. To evaluate the diagnostic yield of VCE for overt OGIB with respect to timing of the intervention, diagnostic yield was analyzed according to the times after latest bleeding. The finding of VCE was classified into P0 or P1 (no potential for bleeding or uncertain hemorrhagic potential) and P2 (high potential for bleeding, such as active bleeding, typical angiodysplasia, large ulcerations or tumors). The P2 lesion was found in 106 patients and diagnostic yield of was 39.1% for overt OGIB. Diagnostic yield of VCE to detect P2 lesion was higher when it is performed closer to the time of latest bleeding (timing of VCE between the VCE and latest bleeding: <24 h, 43/63 (68.3%); 1 days, 16/43 (34.9%); 2 days, 18/52 (34.6%); 3 days, 13/43 (30.2%); 4 days, 7/28 (25.0%); 5–7 days, 6/24 (25.0%), and ≥8 days, 4/18 (22.2%); *p_trend_* < 0.001). The interval between the VCE and latest bleeding were categorized into <24 h (n = 63), 1–2 days (n = 95), 3–7 days (n = 95) and ≥8 days (n = 18). Multivariable analyses showed the odds ratio for P2 lesion detection was 4.99 (95% confidence interval, 1.47–16.89) in <24 h group, compared with ≥8 days group (*p* < 0.010). The overall re-bleeding rate for those with P2 lesion was higher than for those with P0 or P1 lesion at the end of mean follow up of 2.5 years. The proportion of patients who underwent therapeutic intervention including surgery, endoscopic intervention and embolization was higher when VCE is performed closer to the time of latest bleeding (*p* = 0.010). Early deployment of VCE within 24 h of the latest GI bleeding results in a higher diagnostic yield for patients with overt OGIB and consequently resulted in a higher therapeutic intervention rate

## 1. Introduction

Obscure gastrointestinal bleeding (OGIB) was defined as GI bleeding of unknown etiology that persisted or recurred after negative initial evaluation using esophagogastroduodenoscopy (EGD) and colonoscopy [1]. It accounted for approximately 5% of all cases of GI bleeding and was considered to have potential small-bowel bleeding [2,3,4,5]. On the other hands, overt GI bleeding presented with evidence of obvious bleeding, either as melena or hematochezia. Thus, overt OGIB meant that patients showed obvious GI bleeding but negative bidirectional endoscopic evaluation.

Video capsule endoscopy (VCE) was noninvasive tool for evaluation of the entire small bowel in 79–90% of patients, with a diagnostic yield of 38%–83% in patients with suspected small-bowel bleeding [6,7,8,9]. Current guideline recommend VCE as a first-line diagnostic modality for potential small-bowel bleeding [10]. Previous several studies revealed that early performance of VCE in patients with overt OGIB enabled to acquire higher diagnostic yield, lead to appropriate therapeutic intervention, and better outcomes, and reduce medical cost [11,12,13,14,15]. However, there were some studies which showed conflicting results [16,17]. Moreover, the optimal timing of VCE application is unclear [17,18,19,20,21].

We investigated whether early performance of VCE provide high diagnostic yield and improve clinical outcomes in patients with overt OGIB.

## 2. Materials and Methods

### 2.1. Study Population

The VCE data performed from 1 January 2007, to 31 December 2016, at Samsung Medical Center, Seoul, Korea were eligible to this study. Inclusion criteria were as follows: (1) the indication for VCE was overt OGIB, and (2) In-hospital patients. Exclusion criteria were as follows: (1) Outpatients, (2) VCE was performed for non-gastrointestinal (GI) bleeding, (3) patients with occult OGIB, (4) incomplete electronic medical record (EMR), or (5) capsule retention. When patients underwent repeated VCE, the first VCE finding was included in the analysis. OGIB is further divided into overt OGIB, defined by a recurrent passage of visible blood (melena, or hematochezia), and occult OGIB, defined by recurrent iron-deficiency anemia and/or recurrent positive fecal occult blood test results. The Institutional Review Board of Samsung Medical Center approved this study (2018-03-088).

### 2.2. VCE Procedure and Findings

All patients signed a written informed consent before VCE. In Korea, patency capsule was unavailable, so patients did not perform patency capsule before VCE. VCE were performed using a PilCam SB^®^ (SB1 and SB2, Given Imaging, Yogneam, Israel) or a MiroCam^®^ (Intromedic, Seoul, Korea). Board-certificated gastroenterologists reviewed and analyzed VCE findings.

Lesions of interest identified by VCE were classified as having a potential for bleeding, or bleeding. Lesions with potential for bleeding were classified using the following three categories: P2 (active bleeding or high potential for bleeding such as angiodysplasia, ulcers, or tumors), P1 (uncertain bleeding potential such as red spots, erosions, polyps, venous ectasia, diverticulum, or submucosal tumors), and P0 (no bleeding potential such as nodules, visible submucosal veins, or diverticula without the presence of blood). A positive VCE finding was defined when P2 lesions detected by VCE could explain the patient’s focus of GI bleeding.

### 2.3. Outcome Measurement

Laboratory findings including hemoglobin (Hb), platelet count, prothrombin time [13] were measured at the time of GI bleeding. Clinical features of enrolled patients were assessed by reviewing EMR, retrospectively. We reviewed age, sex, underlying diseases (liver cirrhosis, end-stage renal disease (ESRD), and coronary artery disease), history of abdominal surgery, and use of anti-platelet drug, anti-coagulant drug, and nonsteroidal anti-inflammatory drugs (NSAIDs). In addition, we assessed timing of VCE (length time between VCE and last GI bleeding; days), transfusion requirements of pack red blood cell (pRBC), the number of GI bleeding episodes, days of hospitalization, treatment for overt OGIB; therapeutic intervention (surgery, endoscopy, or embolization) and conservative management (medication, or close observation), and re-bleeding rate. The timing of VCE were divided into 4 groups: <24 h, 1–2 days, 3–7 days and ≥8 days.

Primary outcomes were diagnostic yield of VCE and therapeutic intervention rate according to timing of VCE. Diagnostic yield of VCE was defined as the percentage of positive findings detected by VCE over the total number of VCEs performed for overt OGIB. Furthermore, therapeutic intervention rate was defined as the percentage of surgery, endoscopic intervention, or embolization over total number of treatments, including medication or observation. Secondary outcome was risk factor for P2 lesions detected by VCE.

### 2.4. Statistical Analysis

Continuous variables were expressed as mean ± standard deviation or median with interquartile range, while categorical variables were presented as absolute values and percentages. Differences between continuous variables were analyzed using unpaired Student’s *t*-test and Mann-Whitney U test while differences between categorical variables were analyzed using χ^2^ test and Fisher exact test as appropriate. A *p* value < 0.05 was considered statistically significant. Multivariable logistic regression analysis was performed to identify risk factor for P2 lesion detected by VCE. All statistical analyses were performed using SPSS software version 27.0 for Windows (SPSS Inc., Chicago, IL, USA).

## 3. Results

### 3.1. Baseline Characteristics of Enrolled Patients

**Figure 1:**
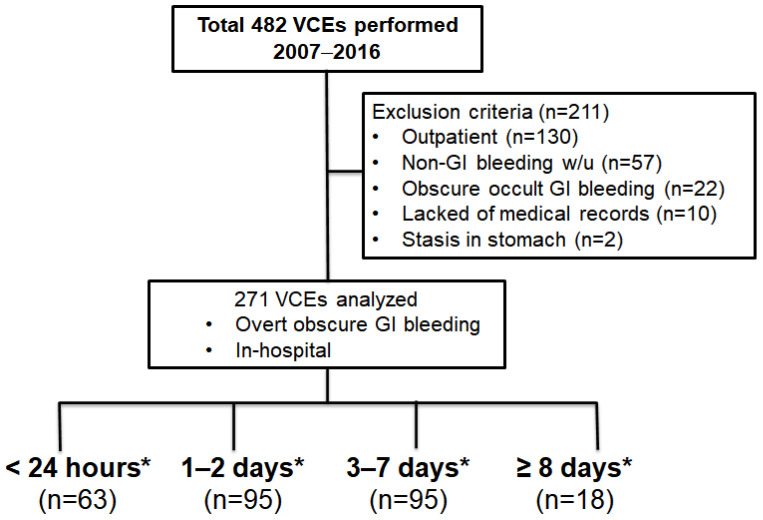
Study flow. * Time interval = VCE − the latest GI bleeding; VCE, video capsule endoscopy; GI, gastrointestinal.

A total 482 sets of VCE performed for overt OGIB from 1 January 2007, to 31 December 2016, at Samsung Medical Center, Korea, Seoul. In this case, 211 patients were excluded and finally 271 patients were eligible in this study (Figure 1).

Comparisons of baseline characteristics of enrolled patients with P2 lesion and with P0 or P1 lesion on VCE finding are shown in Table 1. Mean age of enrolled patients was 61 ± 15.6 years and male were up to 65.7. PT (%) and days of hospitalization in P2 lesion group was longer than P0 or P1 lesion group (*p* = 0.039 and 0.040, respectively). There was no difference in usage of anti-platelet drug, anti-coagulant drug, and NSAIDs and the number of GI bleeding episodes between two groups.

Baseline characteristics according to timing of VCE were shown in Table 2. Platelet was lowest on timing of VCE < 24 h (187.1 ± 67.2 and *p* = 0.010). Transfusion requirements of pRBC was highest and hospital days was longest on timing of VCE < 24 h (5.4 ± 10.0, 13.9 ± 29.6, and *p* = 0.007 and *p* = 0.012, respectively).

### 3.2. Video Capsule Endoscopy Findings

**Figure 2:**
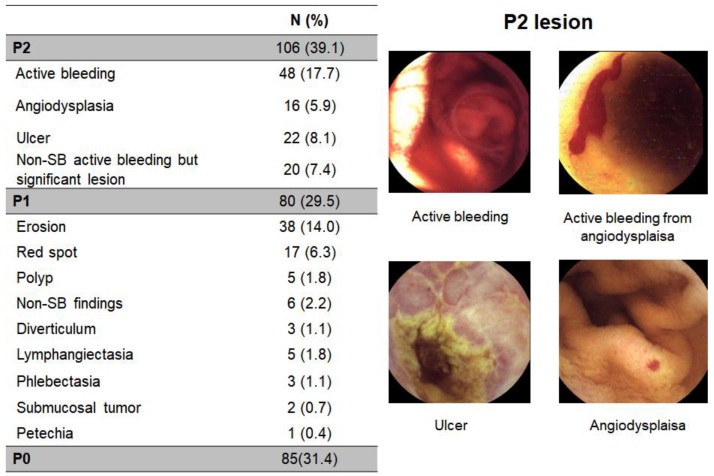
Video capsule endoscopy findings. SB, small bowel.

VCE was able to detect P2 lesions in 106 patients with 39.1% of diagnostic yield. Among P2 lesions, active bleeding was the most frequent findings (n = 48, 45.3%), followed by ulcer (n = 22, 20.8%), non-small bowel (SB) active bleeding but significant lesion (n = 20, 18.9%), and angiodysplasia (n = 16, 15.1%) (Figure 2).

### 3.3. Primary Outcome

**Figure 3:**
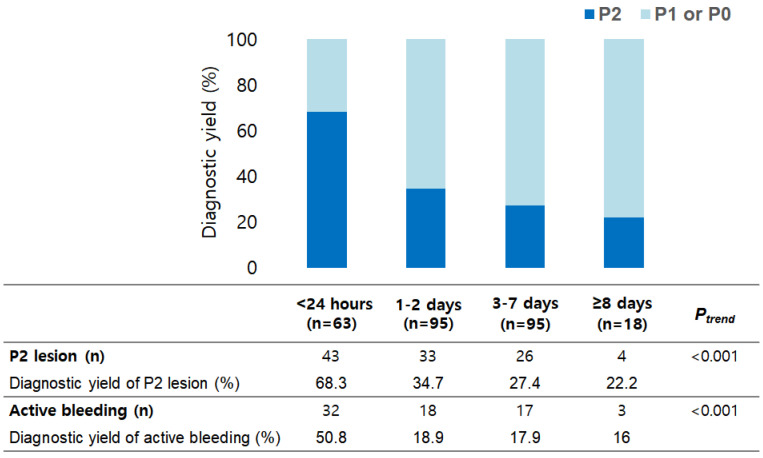
Diagnostic yield to detect and P2 lesion and active bleeding.

Diagnostic yield to detect P2 lesion and active bleeding according to timing of VCE were shown in Figure 3. The detection rate of P2 lesions and active bleeding were decreased significantly with timing of VCE (*p* < 0.001 for both). Therapeutic intervention rate was decreased significantly with timing of VCE (*p* = 0.010) (Table 3). Therapeutic intervention rate for timing of VCE ≤ 1 day was 39.7% (25/63). Therapeutic modalities for timing of VCE ≤ 1 day, were endoscopy in 21 patients, surgery in 3 patients, and embolization in 1 patient.

### 3.4. Secondary Outcome

Risk factors for P2 lesion detected by VCE were shown in Table 4. On multivariable logistic regression analysis, timing of VCE was the risk factor for detecting P2 lesion. The odds for timing of VCE < 24 h were 4.99 times higher, compared to timing of VCE ≥ 8 days (95% CI = 1.47–16.89, and *p* = 0.010).

## 4. Discussion

VCE showed a diagnostic yield up to 83%, but that was affected by the timing of application and the characteristics of bleeding [11,15]. Thus, patients with overt OGIB could acquire the most informative data from VCE and actually they underwent VCE first as diagnostic modality, according to current guideline [10,22]. Early deployment of VCE increased the probability to find out the source or location of GI bleeding. However, there is no consensus on exactly how early to perform VCE and high diagnostic yield of VCE lead to more therapeutic intervention and better clinical outcome.

In our retrospective study about patients with overt OGIB who performed VCE in hospital setting, we demonstrated that the diagnostic yield of VCE for P2 lesion and especially active bleeding decreased as time of VCE was delayed. VCE performed within 24 h of the last overt OGIB after negative bidirectional endoscopic findings achieved a high diagnostic yield in 68.3% of patients, leading to therapeutic intervention in 39.7%.

A notable increase was shown in the diagnostic yield when VCE was applied within 24 h of the last overt OGIB. Detection of P2 lesion and especially active bleeding by VCE declined progressively as day passed after overt OGIB. It seemed reasonable that it was consistent with the natural course of GI bleeding, which spontaneously stopped over time. On multivariable analysis, only risk factor for P2 detection by VCE was timing of VCE < 24 h (OR, 4.99; 95% CI, 1.47–16.89, *p* = 0.010).

Early detection of bleeding focus could lead to prompt therapeutic intervention. Among therapeutic intervention modality carried out for bleeding control in our study, endoscopy was most common (endoscopy, 50; surgery, 14; embolization, 2). Endoscopic treatment was performed the most frequently in VCE within 24 h, compared to other VCE groups (<24 h, 21; 1–2 days, 15; 3–7 days, 12; ≥8 days, 2). Recent systematic review and meta-analysis study revealed therapeutic yield was higher within 2 days after bleeding for small bowel endoscopy (VCE and balloon-assisted endoscopy) and suggested that the optimal timing of endoscopy taking the therapeutic yield consideration would be within 2 days from bleeding [21]. Despite drawback of VCE that could not provide treatment directly, early localization of bleeding lesion by VCE considering its wider availability and noninvasive nature, allowed more patients with overt OGIB to receive proper treatment, especially endoscopic hemostasis, most powerful method with less invasiveness modality and more accuracy to bleeding focus.

Singh et al. and Kim et al. suggested that early performance of VCE within 3 days and 2 days each, associated with reduction of hospital days [18,19]. However, our study was not consistent with them. Our patients who performed VCE within 24 h were hospitalized for the longest time (13.9 ± 29.6 days and *p* = 0.012) compared to other VCE groups. Our study included only patients who performed VCE in hospital setting, in contrast with Kim et al. Furthermore, in subgroup analysis, patients who underwent VCE within 24 h showed the highest transfusion requirement of pRBC (5.4 ± 10.0 and *p* = 0.007). Therefore, there was possibility that patients with more active and severe bleeding perform VCE earlier. As a result, it seemed that early deployment of VCE could not shorten hospital days in VCE < 24 h. Nevertheless, more active and severe cases included into timing of VCE < 24 h group, there was no statistical difference regarding to bleeding related death among four VCE groups (*p* <0.846). From this point of view, we might be able to infer carefully that early performance of VCE contributed to improve patient outcome.

This study had several limitations. First, it was retrospective study. Second, this study was composed of ethnic Korean individuals. Third, this study had an inherent selection bias in that all enrolled patients, especially patients who performed within 24 h had high-risk for active and severe bleeding (higher rate of transfusion pRBC and longer hospital days). Fourth, there are several inherent drawbacks of VCE, including a lack of therapeutic capability. In spite of this, our study revealed that high diagnostic yield and therapeutic rate by early performance of VCE. Fifth, we could not demonstrate improvement of objective indicator for clinical outcome such as hospital days.

VCE performed within 24 h of the last overt OGIB after a negative bidirectional endoscopic finding achieves a high diagnostic yield in 68.3% of patients, leading to therapeutic intervention in 39.7%. These results indicated that VCE might play a crucial diagnostic role when performed close to the onset of overt OGIB. Performing VCE within 24 h could improve management of these patients by allowing for a more rapid, and appropriate therapeutic plan.

## 5. Conclusions

VCE within 24 h from the last overt OGIB results in a higher diagnostic yield and higher therapeutic intervention rate. Therefore, VCE application with a 24 h cutoff could improve the outcome of patients. A further prospective study is warranted to confirm these findings.

## Figures and Tables

**Table 1:** Baseline characteristics of enrolled patients with P2 lesion and with P0 or P1 lesion on video capsule endoscopy finding.

	Total (n = 271)	P0 or P1 Lesion (n = 165)	P2 Lesion (n = 106)	*p*
Age (years)	61.5 ± 15.6	62.2 ± 15.1	60.3 ± 16.3	0.338
Sex, male	178 (65.7)	113 (68.5)	65 (61.3)	0.240
Type of VCE, miroCam^®^	187 (68.6)	112 (67.9)	74 (69.8)	0.789
Interval between VCE and last overt OGIB (days)	3.0 ± 5.7	3.1 ± 3.4	2.7 ± 8.1	0.569
Underlying liver cirrhosis	23 (8.5)	14 (8.5)	9 (8.5)	1.000
Underlying ESRD	13 (4.8)	9 (5.5)	4 (3.8)	0.772
Underlying coronary artery disease	34 (12.5)	19 (11.5)	15 (14.2)	0.575
History of abdominal surgery	39 (14.4)	22 (13.3)	17 (16.0)	0.596
Use of antiplatelet drug	96 (35.4)	57 (34.5)	39 (36.8)	0.795
Use of anticoagulant drug	34 (12.5)	26 (15.8)	8 (7.5)	0.059
Use of NSAIDs	23 (8.5)	13 (7.9)	10 (9.4)	0.661
Hemoglobin (g/dL)	9.5 ± 2.2	9.4 ± 2.2	9.7 ± 2.3	0.334
Platelet (/mm3)	202.4 ± 90.0	198.3 ± 92.9	208.6 ± 85.4	0.361
PT (%)	83.9 ± 18.3	81.9 ± 18.9	86.8 ± 17.1	0.039
Transfusion requirement of pRBC	3.2 ± 5.6	2.7 ± 3.1	4.1 ± 8.0	0.096
Number of GI bleeding episodes	1.6 ± 1.5	1.6 ± 1.7	1.4 ± 0.9	0.290
Hospital days	8.6 ± 15.8	6.7 ± 6.8	11.5 ± 23.5	0.040

VCE, video capsule endoscopy; OGIB, obscure gastrointestinal bleeding; ESRD, end stage renal disease; NSAID, non-steroidal anti-inflammatory drug; pRBC, pack red blood cell; GI, gastrointestinal.

**Table 2:** Baseline characteristics of enrolled patients according to timing of video capsule endoscopy.

	<24 h (n = 63)	1–2 Days (n = 95)	3–7 Days (n = 95)	≥8 Days (n = 18)	*p*
Age (years)	62.7 ± 13.5	59.5 ± 16.9	62.1 ± 14.2	63.8 ± 21.4	0.488
Sex, male	40 (63.5)	61 (64.2)	65 (68.4)	12 (66.7)	0.908
Type of VCE, miroCam^®^	41 (65.1)	66 (69.5)	64 (67.4)	15 (83.3)	0.517
Underlying liver cirrhosis	5 (7.9)	8 (8.4)	10 (10.5)	0 (0.0)	0.531
Underlying ESRD	1 (1.6)	3 (3.2)	8 (8.4)	1 (5.6)	0.192
Underlying coronary artery disease	5 (7.9)	9 (9.5)	16 (16.8)	4 (22.2)	0.160
History of abdominal surgery	15 (23.8)	11 (11.6)	10 (10.5)	3 (16.7)	0.095
Use of antiplatelet drug	17 (27.0)	29 (30.5)	41 (43.2)	9 (50.0)	0.068
Use of anticoagulant drug	5 (7.9)	13 (13.7)	14 (14.7)	2 (11.1)	0.619
Use of NSAIDs	5 (7.9)	9 (9.5)	7 (7.4)	2 (11.1)	0.928
Hemoglobin (g/dL)	9.7 ± 2.1	9.8 ± 2.2	9.3 ± 2.3	9.0 ± 2.4	0.366
Platelet (/mm3)	187.1 ± 67.2	199.3 ± 106.8	203.4 ± 78.5	266.6 ± 98.4	0.010
PT (%)	84.5 ± 16.1	84.5 ± 19.4	82.6 ± 20.0	86.8 ± 10.1	0.816
Transfusion requirement of pRBC	5.4 ± 10.0	2.3 ± 2.9	2.8 ± 3.2	2.9 ± 3.1	0.007
Number of GI bleeding episodes	1.5 ± 1.0	1.7 ± 2.1	1.4 ± 0.8	1.4 ± 0.9	0.499
Hospital days	13.9 ± 29.6	5.5 ± 6.0	8.2 ± 8.1	7.7 ± 5.5	0.012
Re bleeding events	18 (28.6)	22 (23.2)	18 (18.9)	5 (27.8)	0.533

VCE, video capsule endoscopy; ESRD, end stage renal disease; NSAID, non-steroidal anti-inflammatory drug; pRBC, pack red blood cell; GI, gastrointestinal.

**Table 3:** Management of patients with overt obscure gastrointestinal bleeding according to the duration between bleeding and video capsule endoscopy.

	<24 h (n = 63)	1–2 Days (n = 95)	3–7 Days (n = 95)	≥ 8 Days (n = 18)	*p*
Therapeutic intervention	25 (39.7)	19 (20.0)	20 (21.1)	2 (11.1)	0.010
-Surgery	3	4	7	0	
-Endoscopy	21	15	12	2	
-Embolization	1	0	1	0	
Conservative management	38 (60.3)	76 (80.0)	75 (78.9)	16 (88.9)	
-Medication	10	19	18	4	
-Close observation	28	57	57	12	

**Table 4:** Risk factors for P2 detected by video capsule endoscopy.

	Univariable Analysis		Multivariable Analysis	
	OR (95% CI)	*p* Value	OR (95% CI)	*p* Value
Timing of VCE				
<24 h	4.23 (1.34–13.34)	0.014	4.99 (1.47–16.89)	0.010
1–2 days	1.38 (0.45–4.22)	0.568	1.63 (0.51–5.23)	0.414
3–7 days	1.14 (0.37–3.50)	0.816	1.24 (0.39–3.94)	0.720
≥8 days	1	0.001	1	0.001
Age (years)	0.99 (0.98–1.01)	0.337	0.99 (0.97–1.01)	0.283
Sex, male	0.73 (0.44–1.22)	0.226	0.64 (0.36–1.13)	0.123
Underlying liver cirrhosis	1.00 (0.42–2.40)	0.999	1.09 (0.40–2.92)	0.872
Underlying ESRD	0.68 (0.20–2.27)	0.530	1.27 (0.34–4.83)	0.722
Underlying coronaryartery disease	1.27 (0.61–2.62)	0.523	1.50 (0.61–3.71)	0.380
History of abdominalsurgery	1.24 (0.63–2.47)	0.536	0.89 (0.40–1.94)	0.761
Use of antiplatelet drug	1.10 (0.66–1.83)	0.706	1.27 (0.63–2.58)	0.506
Use of anticoagulant drug	0.44 (0.19–1.00)	0.051	0.46 (0.18–1.21)	0.115
Use of NSAIDs	1.22 (0.51–2.89)	0.654	1.01 (0.39–2.60)	0.991
Transfusion requirementof pRBC	1.05 (1.00–1.11)	0.076	1.03 (0.97–1.09)	0.296
Number of GI bleedingepisodes	0.89 (0.71–1.12)	0.309	0.88 (0.68–1.13)	0.319

VCE, video capsule endoscopy; ESRD, end stage renal disease; NSAID, non-steroidal anti-inflammatory drug; pRBC, pack red blood cell; GI, gastrointestinal.

## Data Availability

The data presented in this study are available on request from the corresponding author.
